# *Clostridioides difficile* Infection in Kidney Transplant Recipients

**DOI:** 10.3390/pathogens13020140

**Published:** 2024-02-04

**Authors:** UZhe Ding, Lijin Ooi, Henry H. L. Wu, Rajkumar Chinnadurai

**Affiliations:** 1 Department of Renal Medicine, Northern Care Alliance NHS Foundation Trust, Salford M6 8HD, UK; uzhe.ding@nca.nhs.uk (U.D.); lijin.ooi@nca.nhs.uk (L.O.); rajkumar.chinnadurai@nca.nhs.uk (R.C.); 2 Renal Research Laboratory, Kolling Institute of Medical Research, Royal North Shore Hospital, The University of Sydney, Sydney, NSW 2065, Australia; 3 Faculty of Biology, Medicine & Health, The University of Manchester, Manchester M1 7HR, UK

**Keywords:** *Clostridiodes difficile*, infection, solid organ transplant recipients, kidney transplant recipients

## Abstract

*Clostridioides difficile* (*C. difficile*) is a bacterial organism that typically infects the colon, which has had its homeostasis of healthy gut microbiota disrupted by antibiotics or other interventions. Patients with kidney transplantation are a group that are susceptible to *C. difficile* infection (CDI) and have poorer outcomes with CDI given that they conventionally require long-term immunosuppression to minimize their risk of graft rejection, weakening their responses to infection. Recognizing the risk factors and complex pathophysiological processes that exist between immunosuppression, dysbiosis, and CDI is important when making crucial clinical decisions surrounding the management of this vulnerable patient cohort. Despite the clinical importance of this topic, there are few studies that have evaluated CDI in the context of kidney transplant recipients and other solid organ transplant populations. The current recommendations on CDI management in kidney transplant and solid organ transplant recipients are mostly extrapolated from data relating to CDI management in the general population. We provide a narrative review that discusses the available evidence examining CDI in solid organ transplant recipients, with a particular focus on the kidney transplant recipient, from the epidemiology of CDI, clinical features and implications of CDI, potential risk factors of CDI, and, ultimately, prevention and management strategies for CDI, with the aim of providing areas for future research development in this topic area.

## 1. Introduction

Kidney transplantation is a definitive form of kidney replacement therapy, which is a life-saving intervention for those with end-stage kidney disease, offering an opportunity for a transformed outlook post-transplantation with a significantly improved quality of life and overall outcomes [1,2]. A pivotal component of post-transplant care involves immunosuppression to minimize the risk of graft rejection and ensure the longevity and functionality of the transplanted kidney. However, prolonged maintenance immunosuppressive treatment introduces a notable caveat—that kidney transplant recipients become more susceptible to infections.

*Clostridioides difficile (C. difficile)* is a microorganism that primarily affects the colon. The pathophysiology of the *C. difficile* pathogen involves the disruption of the normal balance of the gut microbiota, leading to the overgrowth of this Gram-positive bacterium [3]. The infection is commonly acquired through the fecal–oral route, with transmission facilitated by the ability of *C. difficile* to form resilient spores [3]. The bacterium resides in the intestinal tract, and while colonization may be asymptomatic, the proliferation of toxigenic strains can lead to the manifestation of disease.

The elevated risk of *C. difficile* infection (CDI) among kidney transplant recipients is attributed to various contributing factors. The immunosuppressive regimens that are prescribed to prevent graft rejection may result in the dysregulation of the immune system, which leads to greater susceptibility towards infections. Additionally, frequent exposure to potential infection sources in healthcare settings, something that is commonplace in post-transplant care, further amplifies the risk of infection [4]. Antimicrobial exposure, often a consequence of post-transplant prophylaxis and treatment regimens, contributes to the disruption of the intestinal microbiota and creates an environment for *C. difficile* proliferation.

Recognizing the risk factors for CDI, and the interaction of immunosuppression, dysbiosis, and CDI, will help clinicians better diagnose and treat CDI in this vulnerable population. There are a limited number of studies that have explored CDI in solid organ transplant populations, in particular the kidney transplant population. Ultimately, current guidance on CDI management in kidney transplant populations are frequently extrapolated from data relating to CDI management in the general population.

We provide a narrative review that aims to consolidate the available evidence examining CDI in the solid organ transplant population, with a particular focus on the kidney transplant recipient subset. A scoping literature review search was conducted by U.D, L.O. and H.H.L.W. independently in the following databases: PubMed, ‘Google Scholar’, ‘Web of Science’, ‘Endnote’, ‘EMBASE’, and ‘Medline ProQuest’. The key search terms for study selection encompassed the following: ‘transplant’, ‘transplantation’, ‘kidney transplant’, renal transplant’, ‘kidney transplantation’, ‘renal transplantation’, ‘*Clostridium difficile*’, ‘*Clostridiodes difficile*’, ‘solid organ transplant’, ‘incidence’, ‘prevalence’, ‘epidemiology’, ‘clinical presentation’, ‘symptoms’, ‘risk factors’, ‘treatment’, ‘management’, ‘outcomes’, and ‘prognosis’. A total of 1048 articles initially appeared from our broad search process, in which 65 articles published between January 2000 and December 2023 were reviewed in full.

## 2. The Epidemiology of *C. difficile* Infections in Kidney Transplant Recipients

In a United States national hospital discharge survey study involving 162 million adults over a 5-year period, Keddis et al. [5] concluded that the incidence of CDI is doubled in the CKD population in comparison to the general population, and particularly more prominent amongst those who are undergoing dialysis treatment. When we consider the solid organ transplant patient cohorts, there are diverse trends that were presented in terms of the incidence and prevalence of CDI between different organ transplant populations. Kidney transplant recipients appear to have a greater risk of CDI than the general population, though at lower rates than lung and liver transplant recipients [6,7,8]. In a meta-analysis involving 21,683 solid organ transplant recipients, Paudel et al. [6] noted that the prevalence of CDI in kidney transplant recipients was comparatively lower at 4.7% (compared to the overall estimated prevalence in solid organ transplant recipients of 7.4%). This finding was concurred in another study by Hosseini-Mogghadam et al. [8], where the CDI incidence was found to be the lowest in kidney transplant recipients amongst all the solid organ transplant recipients included. Overall, current reports on the cumulative incidence of CDI in kidney transplant recipients range between 1 and 8% [4,9,10,11].

Typically, there is an early onset of post-transplant CDI observed amongst kidney transplant recipients. Numerous studies have described the reasons for an acute onset of CDI following kidney transplantation as being attributed to a patient’s vulnerability to CDI from increased exposure to antibiotics and hospital setting exposure as an inpatient and the increased immunosuppression administered during the peri-transplant period [7,8]. A single-center observational study by Neofytos et al. [4] noted that 73% of kidney transplant recipients eventually developing CDI had developed CDI within 30 days of their operation [4]. A larger single-center observational study from Canada involving 1816 kidney transplant recipients by Li et al. [11] reported that most CDI-affected patients would be diagnosed with CDI within 6 months post-transplantation. Otherwise, the multi-national prospective study by Blumberg et al. [7] evaluating CDI in 132 solid organ and hematopoietic stem cell transplant recipients showed a median of 20 days from transplant to CDI. It should be acknowledged that these findings may be influenced by factors such as testing bias, in which physicians may potentially be more likely to order stool cultures at an early phase following transplantation when patients are more vulnerable clinically.

## 3. Clinical Features and Implications of *C. difficile* Infection in Kidney Transplant Recipients

In the United States, the diagnostic process for determining CDI in solid organ transplant recipients is presently advocated to align with the guidance recommended for the general population, as per the 2017 and subsequently 2021 clinical practice guidelines updated by the Infectious Diseases Society of America (IDSA) and the Society for Healthcare Epidemiology of America (SHEA) [12,13]. Confirmation of CDI entails the detection of clinical manifestations, such as diarrhea and/or toxic megacolon, along with confirming the presence of toxins A/B or a toxin-producing *C. difficile* strain in stool samples and identifying pseudomembranous colitis on colonoscopy. While collection and evaluation of stool samples is the standard diagnostic test for suspected CDI cases, rectal swabs may be considered for non-diarrheal potential cases [12,13]. The challenge of diagnosing CDI in the solid organ transplant population arises from the frequently vague nature of diarrheal symptoms in this cohort and suboptimal assay performance (i.e., the inability to accurately distinguish true infection from colonization using the PCR assays for *C. difficile*) [14]. Diarrhea in these patients tends to originate from a myriad of causative factors, including both infectious and non-infectious causes, making the diagnostic process complex. In fact, non-infectious causes of diarrhea were reported to encompass up to 75% of the overall causes in solid organ transplant recipients [15]. Immunosuppressive medications commonly contribute to the development of diarrheal symptoms. Considering the kidney transplant population specifically, prevalent infectious causes of diarrhea may also include cytomegalovirus (CMV) and norovirus in addition to *C. difficile*.

The symptoms of CDI, triggered by bacterial toxins, usually progress from diarrhea to abdominal pain and then the development of pseudomembranous colitis, which, in severe cases, may manifest into toxic megacolon and can potentially be fatal [3,10]. The severity of CDI is linked to the presence of hypervirulent strains, in particular Ribotype 027. Shah et al. [16] noted that kidney transplant recipients with a *C. difficile* infection are more likely to present with leukopenia at the time of diagnosis. The consequences of *C. difficile* infection in the solid organ transplant population, including the kidney transplant population, are further underscored by a significantly higher rate of colectomy (more than three times) compared to the general population [6,8]. Attributable mortality from CDI is deemed to be approximately 1.5%, according to results from the multi-national prospective study by Blumberg et al. [7]. Otherwise, mortality associated with CDI has been cited to be between 2.3 and 8.5% [14].

The risk of graft loss in solid organ transplant recipients due to CDI is a complication that requires attention, with this being especially evident among kidney transplant recipients. The occurrence of CDI has also been shown to be significantly associated with the risk of developing acute kidney injury (AKI) in kidney transplant recipients as well as other solid organ transplant recipients and the general population [17]. The study by Li et al. [11] has pointed out that the development of CDI post-kidney transplant resulted in a greater likelihood of biopsy-proven acute graft rejection, with an odds ratio of five.

Recurrent episodes of CDI amongst the solid organ transplant population are a noteworthy potential issue, with a prospective multinational study by Blumberg et al. [7] pointing out that CDI recurrence rates at 90-day follow-up since the initial CDI episode were 44% in CMV-affected patients and 13% for those without CMV. Indeed, solid organ transplant recipients confront a substantial risk of mortality following each episode of CDI, which is mostly explained by the extent of tissue injury across multiple organs triggered by systemic inflammatory changes [18]. The occurrence of AKI in recurrent CDI cases has resulted in elevated mortality rates [8,19]. Ultimately, comparing kidney transplant and other abdominal solid organ transplant recipients against thoracic solid organ transplant recipients, the former are less likely to develop AKI requiring acute dialysis post-transplant and less likely to be admitted into intensive care during hospitalization [8]. These are important considerations, given that both are risk factors for post-CDI mortality in the short term [8]. 

## 4. Potential Risk Factors of *C. difficile* Infection in Kidney Transplant Recipients

There is a paucity of studies that pinpoint established risk factors for CDI development in the kidney transplant population. It is currently agreed that many of the risk factors for CDI that were identified in other solid organ transplant cohorts would likely apply to the kidney transplant population as well.

Established demographic risk factors associated with CDI development in kidney transplant recipients include being male and having a longer duration of dialysis treatment prior to undergoing kidney transplantation [11,16]. Otherwise, well-cited risk factors in the context of solid organ transplant recipients are often linked to hospital-acquired CDI, such as disruptions in the gut flora due to antibiotics, proton-pump inhibitor (PPI) use, abdominal surgery and intestinal stasis, and environmental exposures during hospitalization. In terms of antibiotics, β-lactams, including penicillin and cephalosporins, were recognized as the highest-risk antibiotics for CDI development, with recent associations also implicating fluoroquinolones [20,21]. It is notable that the prescription of antibiotics following kidney transplantation is prevalent, particularly due to urinary tract infections [22]. Antibiotic exposure and recent hospitalization prior to transplantation are consistently reported as risk factors for higher CDI risk post-transplantation [14]. Given the stomach’s natural acidity, which serves as a deterrent to the survival of *C. difficile* vegetative forms and impedes the germination of its spore form, PPIs have the potential to disturb the gastrointestinal flora balance, thereby establishing an environment conducive to the colonization of *C. difficile* in the bowel. This, combined with the weakened immune defenses observed in the solid organ transplant population, increases their susceptibility to CDI development [23]. It is interesting, though, that Spinner et al. [24] demonstrated that in the kidney transplant population, the clinical impact of PPIs on CDI risk was negligible between the case and control groups. Surgical interventions in hospitals, in particular major gastrointestinal surgeries and procedures, were found to be a significant risk factor for hospital-acquired CDI, further underpinning the intricate interplay between surgical intervention and CDI susceptibility [8,16,25]. The impact of environmental exposure risks in hospital settings, such as contaminated hospital food or contact with hospital furniture, may also become pivotal in influencing CDI susceptibility for the post-transplant population [18]. A longer length of hospital stay following acute recovery from kidney transplantation was found to be a significant factor in the development of CDI, and these findings were consistent with studies focusing on lung transplantation as well [11,18].

The early timing of CDI development following kidney transplantation is likely attributed to the potential asymptomatic colonization with *C. difficile* before and during the transplant procedure. It has been highlighted that asymptomatic detection of *C. difficile* is prevalent in between 10 and 20% of individuals admitted to the hospital [26]. Community-acquired CDI as a route of transmission in the post-transplant population should be acknowledged [27]. Previous reports also observed a significant association between the occurrence of pre-transplant CDI and an increased risk of post-transplant CDI, which underlines the considerable impact of pre-transplant microbial disruptions upon the post-transplant equilibrium [10].

Other potential risk factors contributing to increased CDI development in kidney transplant recipients include receiving a kidney from a deceased donor and the occurrence of biopsy-proven acute rejection within 12 months [11]. Often, there is a challenging clinical scenario that could potentially be foreseeable here, given that diminished graft function in recipients of deceased donor kidneys requires intensified immunosuppressive therapy for acute rejection management. This will compromise the immune response to *C. difficile* and weaken defenses against other infections, hence increasing reliance on antibiotic use. The combination of immunosuppression and frequent antibiotic exposure in the post-transplant phase collectively positions kidney transplant recipients at a significantly increased risk for severe CDI. Nevertheless, no consistent trends of association were determined between specific induction therapies or immunosuppression regimens and CDI risk [7,28]. The intricate balance between immunosuppression and CDI risk comes to the forefront when examining the role of tacrolimus and mycophenolate mofetil (MMF) in this setting. While immunosuppression regimens containing tacrolimus and MMF are implicated in increasing the risk of non-infectious diarrhea as well, it has been demonstrated by multiple studies that elevated tacrolimus levels, which are linked to decreased interleukin-2 production, may compromise immune responses, therefore heightening vulnerability to CDI [24,29,30]. Histopathological findings from intestinal mucosa biopsies of MMF-treated patients displayed inflammatory alterations—crypt cell apoptosis, atrophy, abscesses, and erosion—which can lead to colitis, chronic diarrhea, and cause gut dysbiosis [31].

Adding to the complexity of this clinical scenario following kidney transplantation, the interaction and impact of concomitant viral infections on CDI risk also require specific consideration. Examining the associations between CDI and graft loss in solid organ transplant recipients, one of the few studies that explored this topic by Cusini et al. [7] highlighted that concurrent viral infections, including CMV, in patients diagnosed with CDI did not escalate further risks of graft loss. Although post-transplant viral infections such as CMV did not emerge as a significant contributor to graft loss, the potential influence of CMV on the dynamics of CDI remains a compelling area of study in which more exploration is needed [24].

## 5. Prevention and Management Strategies for *C. difficile* Infection in Kidney Transplant Recipients 

Preventing and managing CDI in kidney transplant recipients presents distinctive challenges for clinicians. This is primarily due to the absence of a fully established prophylactic strategy against *C. difficile*.

Preserving gut microbial diversity is a key aim of the prevention strategy, given that alterations in gut microbial diversity facilitate the environment for CDI onset. Antimicrobial stewardship plays a critical role in preserving gut microbial diversity [12,13,14]. At a broader scale, interrupting *C. difficile* cross-transmission is achievable through strict adherence by clinical staff and patients to hand hygiene practices at all times, i.e., frequent handwashing with soap and water, and implementing effective contact precautions for clinical staff such as using disposable gloves and an apron in clinical areas [12,13,14,32,33]. Additionally, measures to optimize environmental disinfection are essential. In diagnosed CDI cases, isolation measures become crucial to limit the spread of infection [12,13,14,32,33]. Furthermore, prevention of CDI in kidney transplant recipients also entails meticulous attention to adverse effects from pre-existing drugs, particularly immunosuppressive medications such as tacrolimus. Spinner et al. [24] highlighted a 25% elevated risk for CDI development with every 1 ng/ml increase in tacrolimus trough levels. As a precautionary measure, Spinner et al. [24] recommended avoiding routinely maintaining recipient troughs at the higher end of the range unless there are specific concerns in relation to graft rejection.

Investigations are ongoing to determine whether vaccination could be utilized for the prevention of primary and recurrent CDI [34,35]. Another option is to consider bezlotoxumab, a monoclonal antibody targeting *C. difficile* toxin-B that is approved to reduce CDI recurrence and has been recommended by the 2021 IDSA guidelines as an adjunct treatment option where feasible [13]. Gerding et al.’s [36] trial confirmed its efficacy in diminishing recurrent CDI, CDI-related hospital readmissions, and indications for fecal microbiota transplantation (FMT). There remain research gaps currently regarding its use in solid organ transplant recipients.

In terms of managing diagnosed CDI cases in kidney transplant recipients, evidence on the efficacy of oral vancomycin in treating CDI in solid organ transplant recipients is presently lacking. However, current data advocate the use of oral vancomycin as the preferred therapy in cases of severe CDI in solid organ transplant recipients [37,38]. When compared to oral metronidazole, oral vancomycin cured 85–97% of patients versus 65–76% with oral metronidazole [37,38]. There were no differences in clinical outcomes between vancomycin and metronidazole for mild-to-moderate CDI [37,38]. The use of fidaxomicin, a novel macrocytic antibiotic, has emerged with greater popularity in recent years for CDI. It is an alternative antibiotic with a comparable clinical response to oral vancomycin but has been shown to result in a lower CDI recurrence rate [39,40]. Moreover, fidaxomicin has demonstrated minimal interaction with kidney function, as plasma concentrations remain consistently low [41]. Additionally, fidaxomicin is believed to disturb bowel flora less in comparison to other antibiotic agents while also reducing clostridium spore production [39,42]. A consensus recommendation in regard to the duration of antibiotic administration specifically for kidney transplant recipients with CDI remains unestablished, with a lack of data determining whether this should differ with other solid organ transplant recipients and non-transplant patients. Not much is known regarding the risks of frequently relapsing CDI when comparing the solid organ transplant and non-transplant patient groups. There is no consensus regarding a specific management strategy to manage these risks, and there will undoubtably be practice variations worldwide. At present, it is up to a clinician’s holistic assessment of their patient to decide upon the treatment strategy. 

Ultimately, in instances of severe CDI, treatment with antibiotics alone may not be adequate, and consideration of surgical intervention may be necessary. Dallal et al. [43] observed among 78 lung transplant recipients with CDI that 13% of them had to undergo colectomy eventually. It is suggested that colectomy is usually the only life-saving measure in solid organ transplant recipients with fulminant CDI, especially if this is performed prior to the onset of septic shock, where fatality is high [8,44,45].

Otherwise, intensified monitoring of immunosuppression trough levels (e.g., tacrolimus), which can rise during diarrheal episodes, may be helpful to determine appropriate immunosuppression dosing. In severe CDI cases, there should be consideration of temporarily pausing doses with specialist consultation where appropriate. Given the risks of AKI with ongoing diarrhea, adequate volume support to reduce the risk of AKI development is essential.

The utilization of FMT in transplant patients has been a subject of limited exploration due to potential concerns about additional infections. Despite these concerns, FMT has shown established efficacy in managing CDI among other immunocompromised individuals [13,46]. Although incidents involving *Escherichia coli* transmission have raised serious safety concerns, including death, it is crucial to note that FMT can still be a valuable option for treating recurrent CDI in transplant patients who have not responded to antibiotic therapies, emphasizing the need for rigorous donor and specimen screening protocols [46]. Shogbesan et al. [47] conducted a meta-analysis involving 303 immunocompromised individuals with CDI who underwent FMT treatment, reporting that 87% achieved clinical resolution after the initial FMT treatment and that the incidence of adverse effects was comparable to that observed in immunocompetent patients. In another retrospective study, Cheng et al. [48] revealed promising outcomes following FMT treatment in both immunocompetent and immunosuppressed patients, indicating a 64% success rate in resolving recurrent or fulminant CDI with a single FMT. The safety of FMT for immunosuppressed patients has been considered acceptable in this study. Further research is anticipated to determine the safety and efficacy of using FMT to manage CDI in solid organ transplant recipients, including kidney transplant recipients. 

Probiotics have demonstrated promise in the management of CDI in kidney transplants and other solid organ transplant recipients. Dudzicz et al. [49] observed a significant decrease in CDI incidence in kidney transplant recipients receiving *Lactobacillus plantarum* probiotic therapy alongside antibiotic treatment. The cessation of probiotics led to a significant increase in CDI incidence, which highlighted the potential protective effect of probiotic therapy in this context [49]. In a phase three, double-blind, randomized, placebo-controlled trial involving 182 patients with recurrent CDI, the use of purified Firmicutes spores (SER-109) after standard antibiotic treatment showed superiority over placebo in reducing the risk of recurrence [50]. While this study was conducted in a general population experiencing recurrent CDI episodes, further research could explore the efficacy of this treatment modality specifically within the kidney transplant recipient population. Deshpande et al. [51] found that *Lactobacillus plantarum* use was associated with a 3-fold decreased risk of CDI recurrence and with no reported invasive Lactobacillus infections in the lung and liver transplant populations. This suggests that probiotic administration may also have a preventative effect on CDI in addition to the management of CDI cases in solid organ transplant recipients.

In the heart transplant population, post-transplant hypogammaglobulinemia has been shown to independently increase the risk of CDI development [27]. In the setting of kidney transplantation, a case-control study by Origüen et al. [52] has demonstrated significant associations between hypogammaglobulinemia and CDI development in 41 kidney transplant recipients. These results prompt the consideration of intravenous immunoglobulin as a treatment option, though further research is needed on the best strategy for its administration for the post-transplant population. Screening for *C. difficile* colonization in asymptomatic individuals is another consideration. While this measure has been trialed for bone marrow transplant recipients, further work on it in relation to solid organ transplant recipients is needed [53].

In summary, it is encouraging that the ever-evolving therapeutic landscape, from fidaxomicin to more recent innovative approaches such as FMT, offers new strategies for managing CDI in the kidney transplant population.

## 6. Conclusions

**Figure 1:**
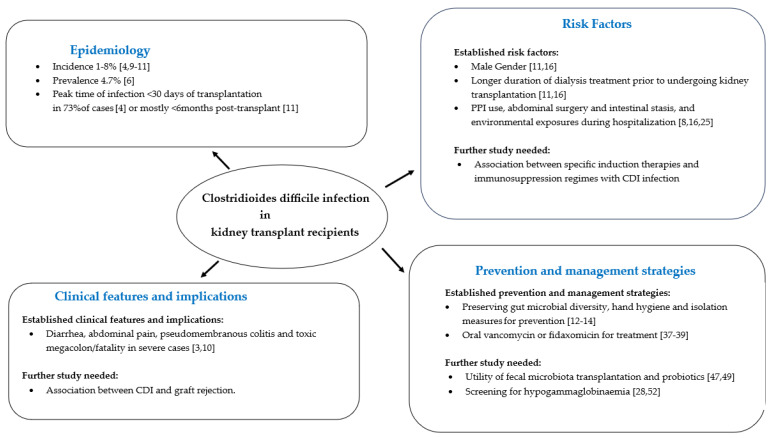
Overview of *Clostridioides Difficile* infection in kidney transplant recipients. CDI: *C. difficile* infection; PPI: proton pump inhibitor.

A greater understanding of *C. difficile* and CDI and improved strategies to manage CDI in kidney transplant and other solid organ transplant recipients requires a multi-disciplinary effort and demands a holistic approach. Figure 1 summarizes our current understanding of this subject and areas for future development. Prospective basic science studies are needed to unravel the intricate relationship between CDI, microbiome changes, and immune response, aiming to provide greater knowledge on the underlying mechanisms. The ultimate goal is to offer translational insights to develop targeted interventions that would specifically safeguard allograft function in kidney transplant recipients. A proactive approach to CDI prevention in kidney transplant recipients should revolve around restricting antibiotic use and adopting effective diagnostics for early detection of CDI. The continuous modification and enhancement of diagnostic tools and the evolution of therapeutic options such as FMT and probiotics tailored to kidney transplant recipients will play a crucial role in managing CDI cases effectively and improving clinical outcomes for this patient population.

## Data Availability

Not applicable.
